# Involvement of the GABAergic Septo-Hippocampal Pathway in Brain Stimulation Reward

**DOI:** 10.1371/journal.pone.0113787

**Published:** 2014-11-21

**Authors:** Germán Vega-Flores, Agnès Gruart, José M. Delgado-García

**Affiliations:** Division of Neurosciences, Pablo de Olavide University, Seville, Spain; University Medical Center Goettingen, Germany

## Abstract

The hippocampus is a structure related to several cognitive processes, but not very much is known about its putative involvement in positive reinforcement. In its turn, the septum has been related to instrumental brain stimulation reward (BSR) by its electrical stimulation with trains of pulses. Although the anatomical relationships of the septo-hippocampal pathway are well established, the functional relationship between these structures during rewarding behaviors remains poorly understood. To explore hippocampal mechanisms involved in BSR, CA3-evoked field excitatory and inhibitory postsynaptic potentials (fEPSPs, fIPSPs) were recorded in the CA1 area during BSR in alert behaving mice. The synaptic efficiency was determined from changes in fEPSP and fIPSP amplitudes across the learning of a BSR task. The successive BSR sessions evoked a progressive increase of the performance in inverse relationship with a decrease in the amplitude of fEPSPs, but not of fIPSPs. Additionally, we evaluated CA1 local field potentials (LFPs) during a preference task, comparing 8-, 20-, and 100-Hz trains of septal BSR. We corroborate a clear preference for BSR at 100 Hz (in comparison with BSR at 20 Hz or 8 Hz), in parallel with an increase in the spectral power of the low theta band, and a decrease in the gamma. These results were replicated by intrahippocampal injections of a GABA_B_ antagonist. Thus, the GABAergic septo-hippocampal pathway seems to carry information involved in the encoding of reward properties, where GABA_B_ receptors seem to play a key role. With regard to the dorsal hippocampus, fEPSPs evoked at the CA3-CA1 synapse seem to reflect the BSR learning process, while hippocampal rhythmic activities are more related to reward properties.

## Introduction

It is generally accepted that hippocampal mechanisms are involved in novelty detection, attention, spatial navigation, and associative learning [1]–[4]. However, little information is available about hippocampal mechanisms involved in the processing of reward, although there is general agreement regarding the involvement of hippocampal synapses in specific associative learning tasks. For example, changes in fEPSPs recorded at the CA3-CA1 synapse have been associated with the acquisition and/or execution of different types of associative learning task [5]–[9]. Another well-accepted mechanism is the involvement of hippocampal rhythmic activities in learning processes, although changes in the different frequency bands (mainly theta and gamma), and their relationships with the observed behaviors, are still under debate [10]–[12].

At the same time, the septal area has been classically described as a rewarding zone able to support BSR with stable characteristics [13]–[16]. Anatomically, it is well described that the medial septum sends (mainly) GABAergic and cholinergic projection fibers to all areas of the hippocampal formation [17]–[25], but probably the projection mainly involved in hippocampal rhythmic activities is that of the septal GABAergic cells [11], [26]. Nevertheless, functional relationships between septo-hippocampal GABAergic projections and the nature of the neural information that they transmit remain poorly characterized.

The hippocampus is a structure related to cognitive processing that could drive animal performance during positive rewarding behaviors. In turn, the involved behaviors are strongly determined by their rewarding value, although not much is known about hippocampal mechanisms that may be related to the neural processing of these rewarding values. Furthermore, medial septum BSR could exert its rewarding effect on the hippocampus through the GABAergic septo-hippocampal pathway.

In order to address all of the above contentions, mice were implanted with stimulating electrodes in Schaffer collaterals of the right dorsal hippocampus and with recording electrodes in the ipsilateral hippocampal CA1 area. Animals were also implanted with stimulating electrodes in the medial septum for BSR. To determine the preferred frequency of medial septum stimulation, animals were trained with a two-choice frequency reinforcement preference task. We used this procedure to determine the effects of different frequencies, with different rewarding values, on the power spectra of LFPs. In subsequent experiments, animals received intrahippocampal injections of selected cholinergic- and GABA_B_-receptor agonists and antagonists to determine their involvement in the acquisition of self-stimulation behaviors.

## Methods

### Animals

Experiments were carried out with mature (6-month-old, 24–35 g) male C57BL/6J mice, obtained from an official supplier (University of Granada Animal House, Granada, Spain). Upon arrival at the Pablo de Olavide Animal House (Seville, Spain), animals were housed in shared cages (5 per cage), but were switched to individual cages after surgery. Mice were kept on a 12 h light/dark cycle with constant ambient temperature (21.5±1°C) and humidity (55±8%), with food and water available *ad libitum*.

Mice included in this study were divided in three groups. i) One group had the complete set of electrodes, consisting of one monopolar electrode for recording from the CA1 area, and two bipolar electrodes for CA3 stimulation and for train stimulation of the medial septum. ii) A second group had a similar set of electrodes, with an additional guide cannula aimed at Shaffer collaterals for drug injection. iii) A third group was split in two: one half had the CA1 recording electrode and the medial septum stimulating electrodes, and the guide cannula, but without the CA3 electrode, to rule out the putative effects of this electrode on LFPs; the other half was implanted with just the CA1-recording and medial-septum-stimulating electrodes, to rule out interference of the cannula in the LFP results. When the animals of these two sub-groups were trained for LFP evaluation in a preference task, no statistical differences were found between them, so they were analyzed as a single group.

We considered successful experimental animals only those that reached all the behavioral criteria and had appropriate electrode placements, as checked histologically. The number of successful animals is indicated in each figure legend. Electrical recordings selected for analysis had to display clear fPSP components in the absence of any sign of epileptiform activity (stimulus-evoked after-discharges, and/or ictal or post-ictal activity), and extracellular recordings (i.e., fPSPs and/or LFPs) that did not deteriorate over time.

### Ethics statement

All experiments were carried out in accordance with the guidelines of the European Union Council (2010/63/EU) and Spanish regulations (BOE 34/11370-421, 2013) for the use of laboratory animals in chronic experiments. Experiments were also approved by the local Ethics Committee (Permit Number 01/2012-14) of the Pablo de Olavide University (Seville, Spain).

### Surgery

**Figure 1:**
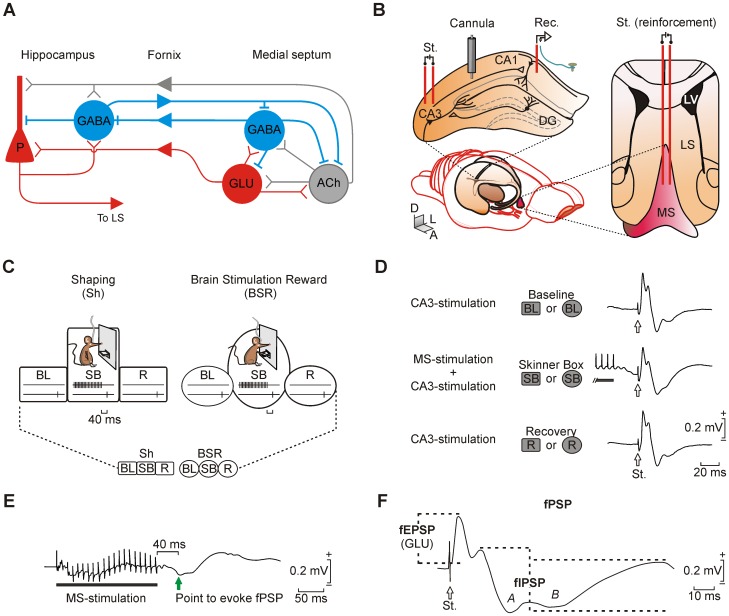
Anatomical septo-hippocampal projections, electrode placement and BSR protocol. (*A*) Schematic representation of the main septo-hippocampal projections. Glutamatergic (red), GABAergic (blue), and cholinergic (gray) projections are indicated. Arrowheads indicate flux direction of neuronal information. (*B*) Animals were chronically implanted with stimulating (St.) and recording (Rec.) electrodes aimed to activate the CA3-CA1 synapse in the right dorsal hippocampus. In addition (right diagram), a bipolar stimulating electrode was implanted in the medial septum (MS). In some animals a guide cannula was also implanted in the dorsal hippocampus. Abbreviations: DG, dentate gyrus; D, L, A, dorsal, lateral, anterior; LS, lateral septum; LV, lateral ventricle; P, pyramidal cell. (*C*) The training protocol to learn brain stimulation reward (BSR) started with some shaping (Sh) sessions. A Sh session consisted of i) a baseline (BL) period for evoking fPSPs at the CA3-CA1 synapse with the animal located in a small box; ii) during a Skinner box (SB) session, the animal was presented with a train of stimuli to the medial septum as reinforcement, followed 40 ms later by a single pulse applied to the CA1-CA3 synapse contingent to approaches to the lever; and iii) a recovery recording (R) period under the same conditions as for BL. After Sh sessions, the animal was allowed to carry out BSR by itself (right). For this, we used the same recording periods (BL, SB, and R) as for shaping. Reinforcements could be received at a maximum rate of one/5 s. At the bottom is shown a diagram summarizing the experimental design, where squares represent the shaping training whilst circles represent BSR protocols. This key diagram is reproduced in the following figures, displaying in dark gray the corresponding stage. (*D*) Illustrative recordings (averaged 10 times) evoked at the CA3-CA1 synapse (arrows) and collected during baseline (BL), 40 ms after a medial septum train (SB), and recovery (R) stages. Examples of how the stage is represented in the following figures by the key diagram are shown. (*E*) Representative recording (averaged 10 times) collected in the CA1 area following train stimulation of the medial septum (black horizontal bar). The green arrow indicates the point where the fPSP will be evoked (40 ms delay from the train). The green arrow indicates the selected moment to evoke an fPSP at the CA3-CA1 synapse. (*F*) Here is illustrated how fPSPs evoked at the CA3-CA1 synapse were divided to compute the amplitude (dashed lines) of the fEPSPs (mediated by glutamate, GLU) and the late fIPSPs. The fIPSP components (A, mediated by GABA_A_ receptors, B, mediated by GABA_B_ receptors) and the stimulus presented to the CA3 area (white arrow) are indicated.

Animals were anesthetized with 0.8–1.5% isoflurane delivered via a mouse anesthesia mask (David Kopf Instruments, Tujunga, CA, USA). The anesthetic gas was supplied from a calibrated Fluotec 5 (Fluotec-Ohmeda, Tewksbury, MA, USA) vaporizer, at a flow rate of 1–2 L/min oxygen (AstraZeneca, Madrid, Spain). Animals were implanted with bipolar stimulating electrodes in the right medial septum (0.1 mm lateral and 0.6 mm anterior to bregma, and 3.8 mm from the brain surface [27]) and in the ipsilateral Schaffer collateral/commissural pathway of the dorsal hippocampus (2 mm lateral and 1.5 mm posterior to bregma, and 1–1.5 mm from the brain surface). A recording electrode was aimed at the CA1 stratum pyramidale (1.2 mm lateral and 2.2 mm posterior to bregma, and 1–1.5 mm from the brain surface). Electrodes were made from 50 µm, Teflon-coated, tungsten wire (Advent Research, Eynsham, UK). A bare silver wire was affixed to the bone as ground. All the implanted wires were soldered to a six-pin socket (RS Amidata, Madrid, Spain) and were then fixed to the skull with dental cement (Figure 1B; see [6], [28]).

For the administration of drugs included in this study, the selected animals were also implanted chronically with a blunted, stainless steel, 26-G guide cannula (Plastic One, Roanoke, VA, USA) in the CA3-CA1 area, close to the hippocampal stimulating and recording electrodes (1.8 mm posterior to bregma, 1.6 mm lateral to midline, and 0.8 mm below the brain surface; [27]). The tip of the cannula was aimed so as to be located ∼0.25 mm above the infusion target. Injections were carried out with a 33-G cannula, 0.25 mm longer than the implanted guide cannula and inserted inside it (Figure 1B).

Animals intended for spectral analysis of hippocampal LFPs were implanted as described above—i.e., some of them (n = 15) without stimulating electrodes in the CA3 area and others (n = 15) without the injecting cannula.

### Electrophysiological recordings and BSR procedures

**Figure 2:**
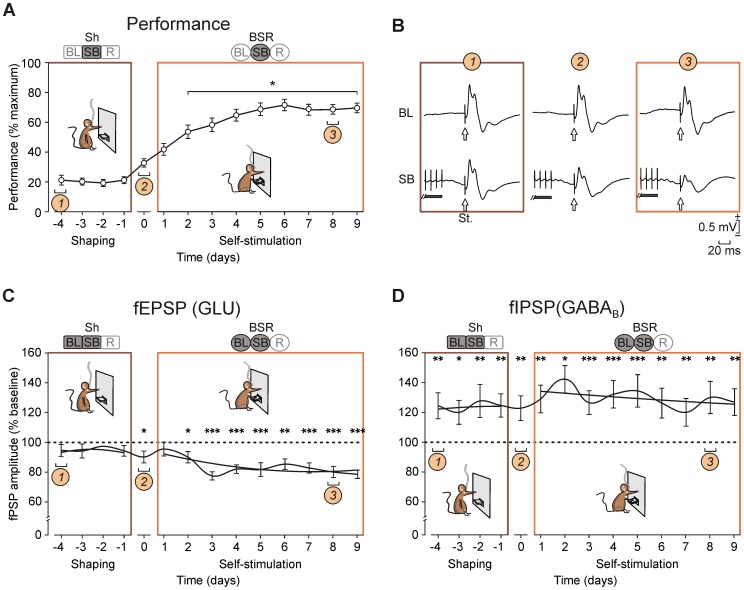
Acquisition of the BSR protocol and changes evoked in fPSPs. (*A*) Animals' performance was computed as (number of reinforcements obtained)/(maximum number of available reinforcements) x 100. Data for each mouse (n = 30) were arranged according to their own zero point, labeled as day “0”. Shaping and BSR are indicated by brown or orange edges, respectively. (*B*) Representative averages (10 times) of fPSPs recorded on three different days during the learning process of BSR. Illustrated fPSPs correspond to the shaping stage (*1*), the day when animals reached BSR criterion (*2*), and eight days after BSR criterion was reached (*3*). White arrows indicate stimulation of the CA3 area (St.). The horizontal black bar indicates a fragment of medial septum stimulation. BL, baseline; SB, recording inside the Skinner box. (*C*) Changes in fEPSP components across training (n = 28). The polynomial trend lines for the amplitude of fEPSP (or GLU) during shaping and BSR stages are indicated. Statistical comparisons are indicated vs. BL values (horizontal dashed line in 100%). (*D*) Changes in fIPSP components across training (n = 28). The polynomial trend lines for the amplitude of GABA_B_ component during shaping and BSR stages are indicated. Statistical comparisons are indicated vs. BL values (dashed line in 100%). (*) *P*<0.05; (**) *P*<0.01; (***) *P*<0.001. Code bars at the top in each section are defined in Figure 1.

**Figure 3:**
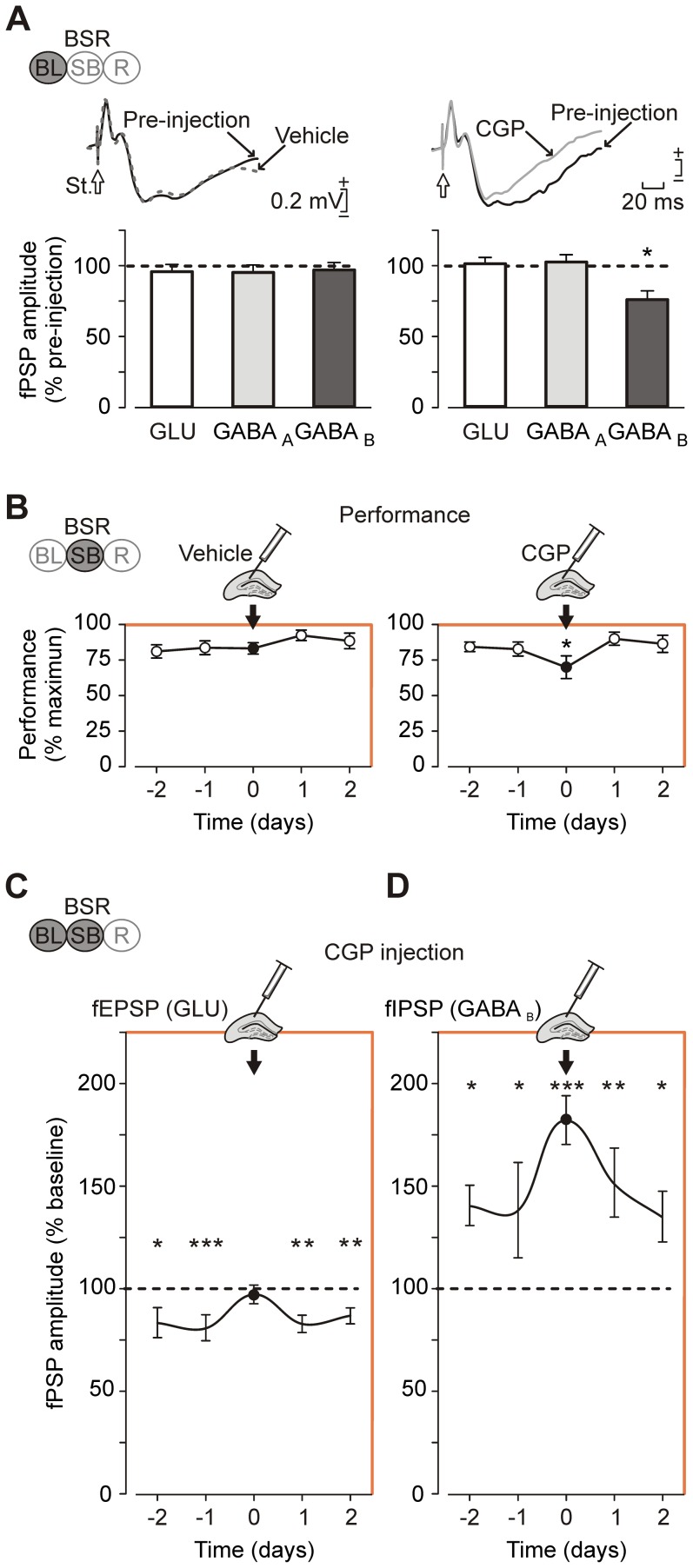
Effects of intrahippocampal injection of CGP 35348 on BSR performance and the associated fPSP changes. (*A*) The upper panel shows representative fPSPs (averaged 15 times) evoked at the CA3-CA1 synapse before injection (black solid line), in the presence of vehicle (gray dotted line) or following CGP injection (gray solid line). The bottom histograms illustrate the averaged fPSP amplitudes corresponding to glutamate- (GLU) and GABA-related components (GABA_A_ and GABA_B_). Comparisons were made vs. vehicle injection (horizontal dashed line). (*B*) CGP effects on animals' BSR determined as (number of reinforcements obtained)/(maximum number of available reinforcements) x 100. Illustrated data range from two days before to two days after (white circles) an intrahippocampal single injection (black circle) of CGP. (*C*) Quantitative effects of CGP injection on fEPSP amplitude. Two sessions prior to (−2, −1) and two sessions after (1, 2) the injection day (black dot) are illustrated. The statistical comparisons are represented vs. BL values (dashed line in 100%). (*D*) As in *C*, same quantitative representation of effects induced by CGP injection but for fIPSP (GABA_B_) amplitude. The statistical comparisons are indicated vs. BL values (dashed line in 100%) (*) *P*<0.05; (**) *P*<0.01; (***) *P*<0.001. Code bars at the top in each section are defined in Figure 1.

Recording sessions started one week after surgery. Field PSP recordings were carried out with Grass P511 differential amplifiers through a high-impedance probe (2×1012 Ω, 10 pF). The electrical stimulus presented to Schaffer collaterals consisted of a 100 µs, square, biphasic, single pulse (Figures 1–3). The evoking stimulus intensity for fPSPs (from 0.02 mA to 0.5 mA) was set usually at 35% of the intensity necessary to generate a maximum fEPSP response [6], [29].

Electrophysiological recordings (shaping and BSR; Figure 1) took place in a Skinner box module measuring 12.5 cm×13.5 cm×18.5 cm (MED Associates, St. Albans, VT, USA) equipped with a lever (or two for the preference test protocol, see below). The shaping (Sh, Figure 1C, D) protocol was carried out as follows: i) The animal was placed for 5 min in a small box (5 cm×5 cm×10 cm) located beside the Skinner box. In this situation, the animal was stimulated at the CA3-CA1 synapse at a rate of 6 stimuli/min, for 5 min, to establish the baseline records (BL, Figure 1C, D); we selected this inter-stimulus interval to rule out paired-pulse facilitation effects. ii) Afterwards, the animal was placed for 20 min in the Skinner box, where it was shaped to press the lever to receive a train of pulses (bipolar, 100 µs pulses presented at 100 Hz for 200 ms, with intensity ≤2 mA) in the medial septum, using a fixed time interval of 5 s (FI5, as described below). This train was followed 40 ms after its end by a single pulse presented at the CA3-CA1 synapse (SB, Figure 1C, D). iii) Finally, the animal was returned to the small box for a recovery period (5 min), during which it was stimulated at the CA3-CA1 synapse at the initial rate of 6 stimuli/min (R, Figure 1C, D). For analysis, fPSPs collected from the CA1 area during the shaping in the Skinner box were compared, using the corresponding baseline values recorded during the same session, as a daily normalization factor for each mouse (Figure 1D).

In accordance with previous reports [30]–[31], during the first two shaping sessions the intensity threshold was adjusted and fixed in some of the animals. The criterion for selecting BSR intensity for each animal was a minimum constant bar pressing in the absence of any observable arrest, general body reaction, or overt movements associated with the presentation of trains of electrical stimulation [28]. The shaping protocol was applied for a maximum of 10 daily sessions and was suspended when the animal reached criterion. The criterion was that the animal performed by itself at least 20 lever presses during a 10-minute period; in addition, this response rate had to present an increasing rate across sessions. It is important to note that a fast starting rate was not possible due to the fixed-time-interval schedule (FI5, as described below). Animals that did not reach the selected criterion during the 10 shaping sessions were eliminated from the study. The BSR protocol was started the day after the selected criterion was reached (Figure 1C, D).

Shaping sessions were followed by several BSR sessions (Figures 1C, D and 2). These were organized as described for shaping sessions, but in this case, train stimulation of the medial septum was carried out only when the animal pressed the lever of its own accord. Figure 2 summarizes the learning process from shaping until BSR. During both shaping and BSR stages, reinforcements could be received at a maximum rate of one/5 s - i.e., with the same fixed-time-interval schedule (FI5). We decided to use this schedule to rule out paired-pulse facilitation effects on the recorded fPSPs. A specific test was carried out to verify this in 7 animals. A single pulse was delivered automatically in the CA3 area every 5 s for more than 30 min to simulate the highest activation of the CA3-CA1 synapse during BSR. The amplitude of the fEPSP was unchanged across this test (*P*<0.952).

### Preference test design

The group of mice used for the preference test had free access to two levers, both delivering 100 Hz as reinforcement frequency during shaping. All mice were trained to use the two levers in an unbiased way at least 3 days before the preference test. To avoid the lever preference side shown by some mice, some sessions with an inactive lever were carried out until lever presses with the two levers were equalized. Only when the animals showed similar BSR performance with both levers did we apply the preference test session. During the preference test, the levers were programmed to deliver two of three frequencies (8 Hz, 20 Hz, and 100 Hz) depending on the experimental design. In accordance with preliminary studies, we chose these three different frequencies of reinforcement to clarify their rewarding effects through a large difference in Hz between trains. These frequencies were tested in the three available permutations, one per day: i) 100 Hz vs. 20 Hz; ii) 100 Hz vs. 8 Hz; and iii) 8 Hz vs. 20 Hz. The order of presentation and day of test was equilibrated among mice. During the preference test session, the relationship between the frequencies that the levers delivered was switched manually with the help of the digital/analog sequencer converter (CED 1401 Plus, Cambridge, England) when the mouse showed clear preference behavior for one lever (∼1 min without change of lever, and ∼5 min with fewer than 10 reinforcements at the “non-preferred” lever). This switching of the reinforcement frequency between levers was carried out as many times as necessary during the session. In order to see clear preference behavior, the preference test was applied from 3 to 4 times per mouse (n = 9 animals) on randomized days. Inside the Skinner box, the schedule, intensity, and lever position remained without change across the whole experiment.

Following a previous report [28], in order to evaluate BSR performance we analyzed different behavioral parameters, such as time spent in pressing the lever, the number of non-rewarded lever presses, and the latency to first reinforcement. However, significant differences were better represented by the relationship (number of reinforcements obtained)/(maximum number of possible reinforcements).

### Drug administration

This part of the study was carried out using an additional group of mice in which the first half of the BSR session was recorded without stimulation in Schaffer collaterals, to collect data for LFP evaluation. In this group, one additional baseline recording was carried out ∼5 min after injection to see online the effect on fPSPs. Only after we observed the expected effect was the animal allowed to start BSR sessions. In order to record all experimental stages within the same time each day, the training time in the Skinner box was reduced to ∼15 min. For intrahippocampal injections, the selected drugs were dissolved in 0.25–0.5 µL of vehicle and injected through the guide cannula at a rate of 0.1 µL/min. Both the GABA_B_-receptor agonist baclofen (90 mM; Sigma-Aldrich, Madrid, Spain) and the selective antagonist CGP 35348 (100 µM; Tocris, Madrid, Spain) were used here. In addition, the cholinergic-receptor agonist carbachol (0.5 mM; Tocris), the M1 muscarinic-receptor agonist McN-A-343 (1 mM; Sigma-Aldrich), and the competitive nonselective muscarinic-receptor antagonist atropine (7 mM; Sigma-Aldrich) were also used. Figure 3 summarizes the data for vehicle and CGP injections. Selected concentrations were initially determined in accordance with previous reports [32]–[36] and adjusted following preliminary tests carried out on implanted mice not included in the reported study.

### LFP recordings

**Figure 4:**
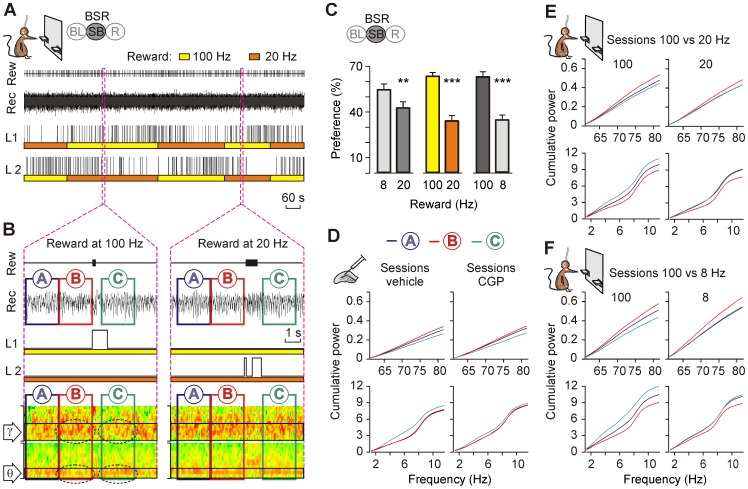
Changes in the spectral power of LFP recorded during animals' BSR. (*A*) A representative preference test session illustrating the comparison between 100 Hz and 20 Hz of BSR with two available levers. From top to bottom are illustrated the obtained rewards (Rew), LFPs recorded (Rec) in the CA1 area, and presses for lever 1 (L1) and lever 2 (L2). Note how the mouse switched levers across the session to receive septal self-stimulation at 100 Hz (yellow) rather than at 20 Hz (red). (*B*) Enlarged sections from *A* for one reinforcement at 100 Hz (left) and one at 20 Hz (right). The LFP channel (Rec.) shows the three time windows (A, blue; B, red, and C, green; each one was 2.2 s long) constructed around each septal self-stimulation. The time that the mouse kept the lever pressed is indicated. The bottom panel illustrates the same recording epochs in a color code for the power spectra. For clarity, only gamma (γ, 60–80 Hz) and low theta (θ, 2–6 Hz) bands are illustrated. Note that the decreased power in gamma and increased power in low theta within window C (dashed ovals) evoked by the reward at 100 Hz were not seen in the reward at 20 Hz. Color scale: green, 100%; red, 200%. (*C*) Preferences in the frequency of reward from the whole group (n = 9). **, *P*<0.01; and ***, *P*<0.001. (*D*) Time windows (A, blue; B, red; and C, green) represented as cumulative power for the group during vehicle and CGP 35348 injections. Gamma (upper) and theta (bottom) bands are shown. The vehicle injection evoked the same changes as the 100 Hz rewards of the preference test (n = 8 animals, 16 sessions). The CGP injection abolished the increased power in the theta band, mimicking the effect of the less-preferred frequencies of reward (8 Hz, 20 Hz) (n = 7 animals, 9 sessions). (*E*, *F*) As in *D*, time windows represented as cumulative power for the group during the preference test. In window C, the less-preferred frequencies of reward (20 Hz, 8 Hz) did not induce changes in gamma and theta bands as the preferred frequency (100 Hz) did. The stimulation with 100 Hz as reward was associated with a decrease in the gamma band and an increase in the theta in comparison with both 20 Hz and 8 Hz (n = 9 animals, 20–30 sessions). Code bars at the top in A and C are defined in Figure 1.

LFPs were recorded from the hippocampal CA1 area in the absence of any electrical stimulation of Schaffer collaterals. To analyze LFP recordings, we defined three time windows around each septal self-stimulation. LFP epochs each lasting 2.2 s were collected in advance of a BSR train (blue A from 4.4 s and red B, from 2.2 s before a BSR; see Figure 4A, B) and from 200 ms after its end (green C, 2.2 s; see Figure 4B). After a visual selection process for artifact- and noise-free epochs, the final power spectrum of each time window average of all the LFPs was calculated. The 200-millisecond delay after reward was aimed at preventing any direct interference of the train response. The frequency analysis is the dominant frequency using the fast Fourier transform (FFT). We normalized the power spectrum data to LFPs using time window B as a reference. The related scripts and analyses of the LFP recordings were developed with the Spike 2 (CED) program. The power spectrum parameters for LFP epochs were 8192 data points (3.7 kHz sampling) for FFT size, 2.2 s length, 0.4521 Hz resolution, Hanning window mode. The data representation for the groups included the analysis of the cumulative power for the different time windows (blue A, red B, and green C) and at the different frequencies of reward (8 Hz, 20 Hz, and 100 Hz) during the preference test (Figure 4E-F) and during the injection of CGP 35348 (Figure 4D).

### Histology

To verify the proper location of implanted electrodes and cannulas, at the end of the experiments mice were deeply anesthetized (sodium pentobarbital, 50 mg/kg) and perfused transcardially with saline followed by 4% paraformaldehyde in phosphate-buffered saline (PBS, 0.1 M, pH 7.4). Their brains were removed and cryoprotected with 30% sucrose in PB. Coronal sections (50 µm) were obtained with a sliding freezing microtome (Leica SM2000R, Nussloch, Germany) and stored at −20°C in 30% glycerol and 30% ethylene glycol in PB until used. Selected sections including the implanted sites were mounted on gelatinized glass slides and stained using the Nissl technique with 0.1% toluidine blue to determine the location of stimulating and recording electrodes and/or the implanted cannula.

### Data collection and analysis

LFPs, fPSPs, 1-volt rectangular pulses corresponding to lever presses (one channel for each lever), and two marker channels (for the single-pulse stimulation of the CA3-CA1 synapse and medial septum train stimulation) were stored digitally on a computer through an analog/digital converter (CED 1401 Plus). Data were analyzed off-line for quantification of animal performance in the Skinner box, LFPs, and fPSPs, using the Spike 2 (CED) program and the video capture system. The amplitude (i.e., the peak-to-peak value in mV during the rise-time period) of 3–5 successively evoked fPSPs was computed and stored for later analysis. These computed results were processed for statistical analysis using the SigmaPlot 11.0 package (SigmaPlot, San Jose, CA, USA). Unless otherwise indicated, data are represented as the mean ± SEM. Acquired data were analyzed with the two-tailed Student's t test or the one-way or two-way ANOVA, mainly with days as repeated measure and with a contrast analysis for a further study of significant differences. For two-way ANOVA, the F_[(m-1), (m-1) × (n-1), (l-m)]_ statistics are shown, with the corresponding degrees of freedom accompanying the F statistic values, where m is the number of orders, n the number of mice, and l the number of multivariate observations [37]–[38].

## Results

### fPSPs evoked at the CA3-CA1 synapse of alert behaving mice

In a preliminary set of experiments we determined the profiles of fPSPs evoked in the CA1 area by the train stimulation of the ipsilateral medial septum, in absence of Schaffer collateral stimulation. As illustrated in Figure 1E, train stimulation of the medial septum evoked a negative-positive (0.27±0.02 mV, peak-to-peak) extracellular field potential in the hippocampal CA1 area with a latency to reach the negative peak of 43±1.5 ms and a duration of 190±10 ms.

In accordance with a recent report [28], electrical stimulation of the CA3-CA1 synapse was presented 40 ms after the end of manual stimulation (shaping) or self-stimulation (BSR) of the medial septum (Figures 1D, E and 2B). This is the delay that introduced the largest changes in the amplitude and profile of fPSPs evoked at the CA3-CA1 synapse. Those changes will be described in detail in the following section. As described previously [1], [39]–[41], fPSPs evoked in the CA1 area by electrical stimulation of Schaffer collaterals presented three components: one with a positive phase due to activation of glutamate receptors, and two subsequent negative components corresponding to the successive activation of GABA_A_, and GABA_B_ receptors, respectively (Figure 1F). The mean latencies for these three successive components were 3.5±1.25 ms (range 2.25–5 ms) for glutamatergic receptors, and 13.5±0.9 ms (range 12–15 ms) and 30.3±4.3 ms (range 26–36 ms) for GABA_A_ and GABA_B_ receptors, respectively.

### Acquisition of BSR and modulation of fPSPs evoked at the CA3-CA1 synapse

Animals were firstly shaped to associate lever presses with train stimulation of the medial septum. For this, a daily session of 20 min maximum was carried out for each animal (Figure 2A). As a success criterion, animals were required to press the lever a minimum of 20 times during a 10-minute period. Animals failing to reach this criterion in ≤10 days were eliminated from the study. Once the criterion was reached, mice were allowed to self-stimulate (i.e., BSR). The animals' performance in the Skinner box during BSR is illustrated in Figure 2A (n = 30). As shown, the percentage of self-stimulations increased during the first 5 sessions until reaching asymptotic values (≈70% of the maximum possible values). When compared with the shaping stage, BSR rates reached significantly (*P*<0.001) higher values from the 2nd to the 9th sessions (Figure 2A).

Both shaping (i.e., manual stimulation) and BSR (self-stimulation) of the medial septum modified the amplitude of fPSPs evoked in the CA1 area (Figure 2B-D). Interestingly, the effects of medial septum BSR were significantly [F_(13,351,783)_ = 4.652; *P*<0.001] greater than those evoked during shaping, specifically for the glutamatergic component (fEPSP, Figure 1F). In contrast, the GABA_B_ [F_(13,351, 783)_ = 4.160; *P*<0.001] components (fIPSP, Figure 1F) were significantly larger from the first shaping session on (Figure 2B-D). Finally, the GABA_A_ component presented a similar trend to that of the glutamatergic component [F_(13,351,783)_ = 3.099; *P* = 0.005; not illustrated]. As shown in Figure 2C, the glutamatergic component (i.e., the fEPSPs; Shaping, y = −0.9325x^2^+4.6626x+90.384; r^2^ = 0.75; BSR, y = 0.343x^2^–4.8347x+96.88; r^2^ = 0.55; *P*<0.001) presented a decreasing trend during the BSR stage. In contrast, the late component of fIPSPs, evoked by the activation of the GABA_B_ receptors (Figure 2D), was not significantly (Shaping, y = −0.2651x^2^+1.9589x+120.55; r^2^ = 0.05; BSR, y = 0.0356x^2^−1.4459x+135.78; r^2^ = 0.23; *P*<0.001) modified during BSR sessions.

### Contribution of glutamatergic, GABAergic, and cholinergic receptors to the proper performance of BSR

In an additional set of experiments, we studied the specific contribution of hippocampal glutamatergic, GABAergic, and cholinergic receptors to the performance of septal BSR. As already indicated, medioseptal axon terminals in the hippocampus include three types (glutamatergic, GABAergic, and cholinergic) of projection fibers, although the function—and even the existence—of the glutamatergic projection is still under debate [18], [20], [22]–[25].

In concurrence with a recent study [28] on the involvement of septo-hippocampal GABAergic projections in septal BSR, we carried out a pharmacological study of the specific role of hippocampal GABA_B_ receptors in the performance of BSR. By means of the fPSP recordings we were able to corroborate the expected effect of drug administration on the fEPSP and fIPSP components evoked by the activation of the CA3-CA1 synapse. Previous studies [32]-[33] have reported that the administration of CGP 35348, a GABA_B_ antagonist, does not have any noticeable effect on fEPSPs. As illustrated in Figure 3A, the administration of CGP 35348 evoked a decrease in the amplitude of only the late fIPSP, corresponding to the GABA_B_ receptor, as compared with values collected for vehicle injections (*P* = 0.015).

Interestingly, CGP 35348 administration evoked a significant (*P*<0.017) decrease in BSR performance (Figure 3B). In addition, an analysis [F_(8,48,104)_ = 3.649; *P* = 0.002] of CGP 35348 effects on fPSPs evoked in the CA1 area during BSR indicated a significant increase in the fEPSP (*P*≤0.05, Figure 3C) and fIPSP (*P*<0.001, Figure 3D) amplitudes. Taken together, these results support the differential involvement of hippocampal GABA_B_ receptors during the performance of BSR, with specific effects on performance efficiency and on the amplitudes of fEPSPs and fIPSPs (GABA_B_ components) evoked by single-pulse activation of the ipsilateral Schaffer collaterals.

We also considered here the putative role of other drugs on BSR and on the concomitantly recorded fPSPs. Thus, the intrahippocampal administration of the GABA_B_ agonist baclofen decreased fEPSP amplitude (*P*<0.001). Atropine (a competitive nonselective antagonist of muscarinic receptors) increased (*P*≤0.05) the amplitude of fIPSPs (for both GABA_A_ and GABA_B_ receptors) evoked in the CA1 area, while the administration of McN-A-343 (an M1 muscarinic receptor agonist) and of carbachol (a cholinergic receptor agonist) decreased (*P*≤0.05) the amplitude of both fEPSP and fIPSP (mostly for GABA_A_) components of the fPSP evoked at the CA3-CA1 synapse. Interestingly, these drugs did not evoke any significant change in BSR performance (data not illustrated), and were not further considered in the present study.

### Two-choice frequency reinforcement preference test

In this two-choice task, mice were presented with two levers, each providing a train of 20 pulses at different frequencies (8 Hz, 20 Hz, and 100 Hz). In simultaneity we recorded LFP in the CA1 area. These electrocortical recordings were carried out in the absence of any electrical stimulation of hippocampal Schaffer collaterals. As illustrated in Figure 4A, and to avoid any undesired conditioning, the frequencies provided by the levers were switched depending on the behavior of the mouse, which was evaluated online. In accordance with previous reports [15]–[16], [42], we verified that the behavioral data satisfied the requirements set to establish that the animal was responding to the reinforcing value. The time spent by the mouse pressing the lever was significantly shorter (*P*<0.05) when the lever was in the time-out period of the FI5 ratio than when the lever press delivered a medial septum train. There was a clear extinction process if both levers were inactivated, as well as a clear reversal in lever preference when the roles of the two levers were switched (Figure 4A).

### Hippocampal LFP activity recorded during BSR

In order to analyze the LFP activity of the hippocampal CA1 area during BSR by using different rewarding frequencies (8 Hz, 20 Hz, and 100 Hz), three LFP epochs (each lasting 2.2 s) were collected for 4.4 s before (time windows A and B) until 2.2 s after (time window C) a reinforcement was obtained by pressing either of the two levers (Figure 4A, B).

In Figure 4E-F is represented the cumulative power analyzed along successive preference sessions, corresponding to the three (A, B, and C) time windows for BSR computed for each lever press. From the whole two-choice session, we selected, for each frequency of reinforcement, a total of 30–40 artifact- and noise-free lever presses. Mice preferred the lever that delivered trains at 100 Hz over those delivering the same number of pulses (i.e., 20) at 20 Hz (*P*<0.05) or 8 Hz (*P*<0.05). In addition, the power spectra of LFPs recorded during time window C (i.e., corresponding to LFPs recorded after the septal BSR) presented significantly (*P*≤0.05) larger spectral powers in the band from 3 Hz to 6 Hz (low theta) than those collected during time windows A and B. Furthermore, the power in time window B (i.e., corresponding to LFPs recorded immediately before the medial septum self-stimulation) was larger, with a frequency of around 9 Hz, but lower in the low theta band (*P*≤0.05) than that corresponding to LFPs recorded in time window A. Interestingly, we found a decrease in the gamma band (60–80 Hz) associated with the preferred frequency of reinforcement: 100 Hz (Figure 4E, F).

The same analysis was applied exclusively to the sessions of intrahippocampal injections of the GABA_B_ antagonist, CGP 35348 (Figure 4D), because this was the only drug disturbing BSR performance. Interestingly, the injection of CGP 35348 reproduced the LFP results in the low theta band, but not in the gamma (Figure 4D).

**Figure 5:**
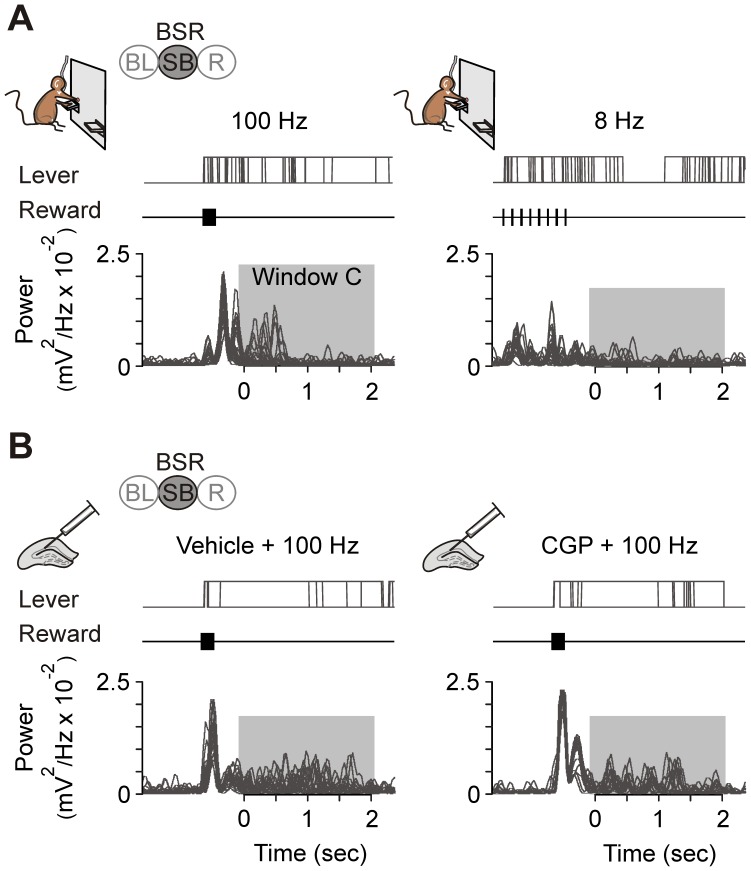
Power spectra in the low theta band evoked by the different reinforcement frequencies and by the local injection of CGP 35348 during time window C, and their relationship with the number of lever presses. (*A*) Power spectra values collected from a representative animal. From top to bottom are illustrated lever presses (Lever), the reward train (Reward), and the power value corresponding to the low theta (3–5.8 Hz) band. Two frequencies of reward tested (100 Hz, 20 Hz) are shown in relation to lever-press activity. Each section (panels 4 s long) corresponds to 30 overlapped sweeps (the lever trace remains high for the time that the lever is held down) as well as their corresponding power values in the low theta band. Gray squares indicate time window C. Note the increase in power spectrum values related to the preferred frequency of reinforcement (100 Hz). This increase was not associated with lever activity. (*B*) Power values collected—always using 100 Hz of reward—from a representative mouse in two different sessions: vehicle and CGP injections. Traces are displayed as in *A*. Note that the increase in power values during vehicle administration sessions was clearly larger than during CGP sessions. Again, no relationship with lever activity was noticed. Code bars at the top in A and B are defined in Figure 1.

A complementary analysis was carried out within window C in order to rule out the possible interaction in these results with a walking state of the recorded mice. Figure 5 shows the power spectra of the low theta band (3 Hz to 6 Hz) in two experimental situations: during the preference test (Figure 5A) and during vehicle and CGP sessions (Figure 5B) associated with the pattern of lever presses. The increased power in the theta band was not associated to locomotor behaviors, because it was present even during repeated lever presses and when the mouse kept the lever pressed for an extended period (even>2 s).

**Figure 6:**
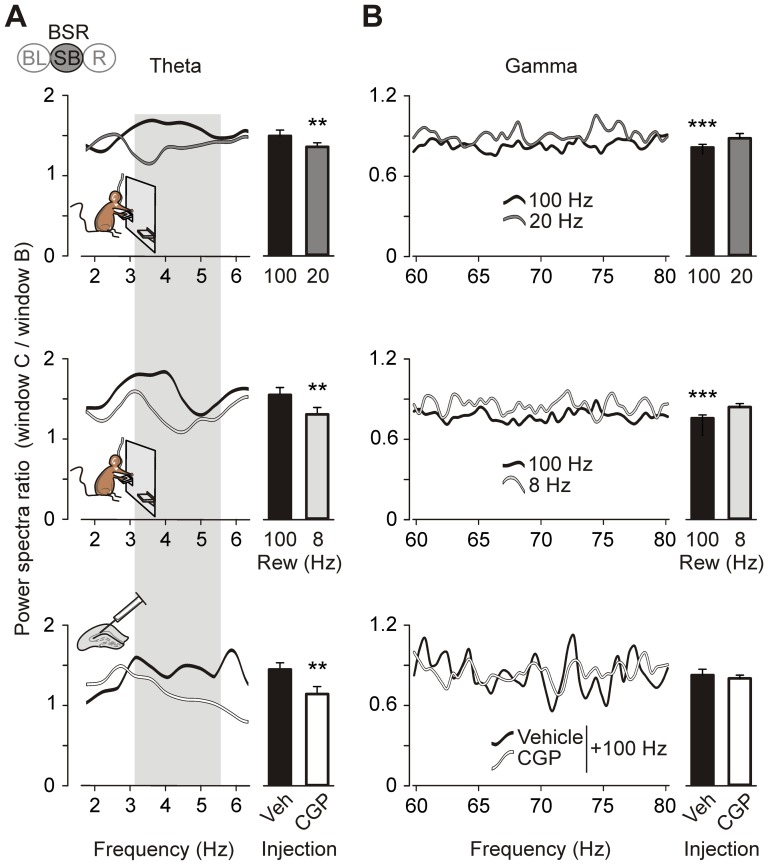
Differences in the ratio time window C (after reward)/time window B (before reward) for power spectra computed from LFP evoked in the hippocampal CA1 area during the preference test and following CGP 35348 intrahippocampal injections. (*A*) Ratio for the low theta band (2–6.3 Hz) for the three frequencies of reward (upper, 100 Hz vs. 20 Hz; middle, 100 Hz vs. 8 Hz) as well as for vehicle vs. CGP injections (bottom). The bars on the right represent the total average for the gray band (3–5.8 Hz). (*B*) The same ratio as in *A*, but represented for the gamma band (60–80 Hz). *, *P*<0.05; **, *P*<0.01; and ***, *P*<0.001. Code bar at the top in A is defined in Figure 1.

Figure 6 illustrates in detail the changes observed in the power of the low theta and gamma bands. These changes were better seen when we compared the ratio between powers collected post-reinforcement (i.e., time window C) and those collected pre-reinforcement (i.e., time window B). The comparison between self-stimulation at 8 Hz and that at 20 Hz (data not shown) did not show any significant difference in the low theta (*P* = 0.074) or in the gamma (*P = *0.093) bands, whereas there was a significant (*P* = 0.004; Figure 6A) increase in the low theta band and a corresponding decrease (*P*<0.001; Figure 6B) in the gamma when septal BSR was performed at 100 Hz. It should also be noted that the increase in the power of the low theta band evoked by trains presented at 100 Hz was canceled out by the intrahippocampal administration of CGP 35348 (*P* = 0.019; Figure 6A, bottom graph and histogram) without any effect on the gamma band (*P* = 0.623; Figure 6B, bottom graph and histogram).

## Discussion

### Role of the GABAergic septo-hippocampal pathway in BSR

Septal nuclei seem to play a role in the integration of internal drives and learning and memory processes [14] due to their nodal functions [22], particularly via the septo-hippocampal pathway [43]. Indeed, it is well known that medial septum stimulation during instrumental conditioning can be rewarding in mice and rats—namely, the animals will generate specific behaviors (lever presses) to obtain this internal reward [13]–[14], [43]–[45]. However, and in agreement with Kaifosh and colleagues [26], the nature of the information carried by the septo-hippocampal circuit associated with the BSR behavior has remained unknown until now. The present results suggest that the information transmitted by the septo-hippocampal GABAergic pathway is essential for both the associative learning and the processing of the reward value, modulating hippocampal mechanisms that probably encode the behaviors involved in BSR.

The GABAergic system has been implicated in emotional displays, motivational states, and the codification of expected reward due to its important modulatory effect on dopaminergic neurons [46]–[49], for example in the ventral tegmental area [50]–[51]. Because that rewarding effect has been classically associated with the dopaminergic system, it is important to point out that dopaminergic fibers are scarcely reported in the septo-hippocampal pathway, if at all. Indeed, some authors have noted that the rewarding effect of septal stimulation may not be fully dependent on a dopaminergic origin [14]. Thus, septo-hippocampal GABAergic and cholinergic fibers could be related to the early processing of the brain stimulation effect. In particular, septo-hippocampal GABAergic neurons terminate on hippocampal basket and axo-axonic interneurons [19], [52], playing a regulatory role in the intrinsic excitability and rhythmic activities of hippocampal circuits [10], [53]–[54]. These septo-hippocampal GABAergic circuits are important in the integration of internal motivational states [16] which in turn modulate cognitive processes characteristically ascribed to the hippocampus [1], [6]–[7], [9]. Recent studies suggest that the deficit of these GABAergic inputs to the hippocampus results in disturbances of septal BSR and hippocampal rhythmicity [28], [52]. Accordingly, and as shown here, the septo-hippocampal GABAergic pathway could play a crucial role in internal rewarding processes.

### The hippocampus and the learning process during BSR

Besides during the shaping period, the effects of BSR on fEPSPs recorded in the CA1 area were inhibitory. Interestingly, when animals had reached the selected criterion, the rate of decrease in fEPSP amplitude was potentiated. The amplitude of the early response of the fIPSP (GABA_A_) showed the same trend as that of fEPSPs, whereas the GABA_B_ component increased from the first shaping session, remaining significantly increased across the whole experiment. These differential effects were observed in most recording sessions, including those of BSR, supporting the notion of a different involvement of the two types of receptor (glutamate vs. GABA_B_) in reward processing.

The hippocampus is involved not only in learning and memory processes, but also in detection [55] and attention phenomena [56]. Some authors claim the CA1 area is “a comparator that computes novelty” [2]. Additionally, it is well known that fPSPs recorded in the CA1 area change in parallel with the acquisition curves of associative learning tasks [1], [6], [7]. In accordance with these contentions, fPSP evolution across training could be due to a stronger hippocampal activity in the early stages of learning than during the final training days. Indeed, fEPSP changes across BSR were significant only after day 0 (i.e., when they reached criterion), suggesting that the hippocampus was resistant to septal inhibition during the initial acquisition stages. For instrumental learning, the first training days involve the highest intentional level [57], [58], when hippocampal activation must be highest and the reward evaluation is most needed, even just for the novelty component [59]. If the CA1 area is acting as a comparator that computes novelty, the shaping days represent the highest level of exposure to novelty. For this reason, it is possible that the inhibition exerted by sepal stimulation on fEPSPs is not as evident during shaping sessions as during the subsequent BSR sessions. Accordingly, the present results suggest that the hippocampus is participating more actively in the early stages of the learning process. Indeed, fEPSP amplitudes decreased more significantly during the later BSR sessions, when the cognitive requirements went down.

### A putative role of hippocampal GABA_B_ receptors in BSR

It has already been reported that the GABA_B_ antagonist CGP 35348 reduces the late fIPSP components at hippocampal synapses [32]–[36]. Here, intrahippocampal injections of CGP 35348 decreased BSR performance, while other behavioral parameters, such as the time that the animal kept the lever pressed, the number of non-rewarded lever presses, or the total number of rewarded and non-rewarded lever presses, remained unchanged. In addition, the decrease in the spectral power density of the low theta band following CGP 35348 sessions mimicked data collected during the preference test. Thus, in the presence of CGP 35348, the stimulation with the preferred rewarding frequency (100 Hz) mimics the decrease in the low theta band evoked by the less-preferred ones (8 Hz and 20 Hz).

### Preferred frequencies for BSR and the related changes in hippocampal LFPs

As already reported [14]–[16], and confirmed here, 100 Hz is the frequency of reinforcement that induces the most-stable BSR behavior with the highest self-stimulation rates. However, a high lever-press ratio is not necessarily related to a higher reinforcement value [30], [46], [60]. In this regard, the two-lever preference test allowed the animal to determine the preferred frequency by direct comparison. With this experimental approach, we were able to evaluate the rewarding effect in a non-time-dependent test for evaluation of rewarding gradients [30]. In addition, the time windows designed for power spectrum computation allowed us to determine LFP changes in relation to the BSR behavior [61]–[62]. Whereas window B could represent the appetitive behavior that drives the animal to the reward and triggers lever-press behaviors, window C probably represents the consummatory behavior where the reward value must be evaluated in order to continue searching for this reward or not.

As a main result, we found an increased spectral power in the low theta band accompanied by a decrease in the spectral power in the gamma band during time window C, using 100 Hz as a rewarding frequency. The other two rewarding frequencies did not induce these changes. Recent studies support a correlation between septal neuronal activity and fast hippocampal rhythms [26]. Lisman and Jensen [12] have proposed that the gamma rhythm codifies information processing in the hippocampus. At the same time, theta oscillations have been called “traveling waves” due to their peculiar propagation across the hippocampal formation. Lubenov and Siapas [63] claim that the transition of these waves is very important for timing and direction of the neural information. Colgin [11] pointed out the importance of coupling mechanisms between theta and gamma frequencies for behaviors associated to reward, memory, and learning. Finally, it has been reported that a decrease in the complexity of GABAergic interneurons, particularly axo-axonic and basket parvalbumin-positive cells [52]—the same kind of neuron involved in the generation of rhythms in the theta and gamma bands [3]–[4], [19], [64]–[67]—is associated with both a diminution in the learning and performance of septal BSR and a decrease in the power of theta and gamma bands [28], [52].

In time window C, our results show that some rewards (8 Hz or 20 Hz) did not decrease the spectral power of the gamma band. These results support the notion of the gamma band as an information encoder, because BSR at 8 Hz and 20 Hz evoked lower reward values than at 100 Hz. Thus, the significant decrease in gamma power for 100 Hz makes sense because this is the expected reward, and the gamma band does not need to encode new information. On the other hand, if the theta rhythm is distributing the information encoded in gamma waves, expected rewards—such as 100 Hz—could be associated with an increased power of the low theta band because this is the expected reinforcement in trained mice. In agreement with Seager [68], our results suggest that sensory information that is already learned can be reflected as higher power in the theta band, and this is facilitating the traffic of information between the medial septum and the hippocampus.

Although we did not analyze in detail the theta band associated with walking activity (7–10 Hz; [69]) (most studies are concerned with the high or the whole theta band, not with the low-frequency component), our results show that changes in the low theta band were not associated with the walking activity of the mouse. Even when the animal keep the lever pressed (i.e., in a situation in which it could not be walking), we were able to find an increase in the low theta band during the window C period.

### Changes in hippocampal LFPs related to septo-hippocampal GABAergic projections during BSR

In time window C, the increased power of the low theta band during the preference task using 100 Hz as reward was pharmacologically canceled out by intrahippocampal CGP 35348 injections. In contrast, the decrease in the gamma band was unaffected. Importantly, of all the drugs tested here, only CGP 35348 injections evoked a decrease in BSR performance similar to that produced by lower (≤20 Hz) rewarding frequencies. This suggests a significant participation of GABA_B_ receptors in BSR performance, probably during the processing of the rewarding value of train self-stimulation. The present results are indicative of the important role of the inhibition induced by activation of GABA_B_ receptors in the processing of the reward at the hippocampal level. In support of this notion, some authors [70] have proposed that the GABAergic system underlies the cognition-enhancing effects of muscarinic receptor activation in the septo-hippocampal pathway. Finally, recent studies performed in the septo-hippocampal GABAergic pathway postulate the GABA_B_ receptor as a very important mediator for appropriate coordination between septal neuron activities and fast hippocampal rhythms [26].

Our study documents in behaving animals that the septo-hippocampal GABAergic system is directly involved in two fundamental aspects of septal BSR that are reflected in the hippocampus as two main mechanisms. The first is the cognitive processing related to the learning process, analyzed through the fPSPs. Thus, the hippocampus is gradually affected by septal stimulation across the successive training sessions. Besides, evoked fEPSPs are resistant to inhibition during the acquisition process, but are inhibited during the late execution of the BSR protocol. This is probably linked to the different cognitive levels required across the learning process. In contrast, the late component of fIPSPs remains significantly increased across all the sessions. This suggests that fIPSPs do not reflect the learning process, as fEPSPs do. The local blockage of GABA_B_ receptors reverted fEPSPs to baseline values, in parallel with a decrease in BSR performance. The second hippocampal mechanism is the rhythmic activity which seems to be involved in the processing of the reward value, specifically in sub-bands of gamma frequencies. Finally, septo-hippocampal GABAergic projections could be related to changes in hippocampal rhythmic activities [3]–[4], [53]–[54], [63], [69], supporting a functional role of gamma rhythms in the processing of the reward value.

### Relationships between fPSP amplitudes and changes in hippocampal rhythmic activities during BSR sessions

**Figure 7:**
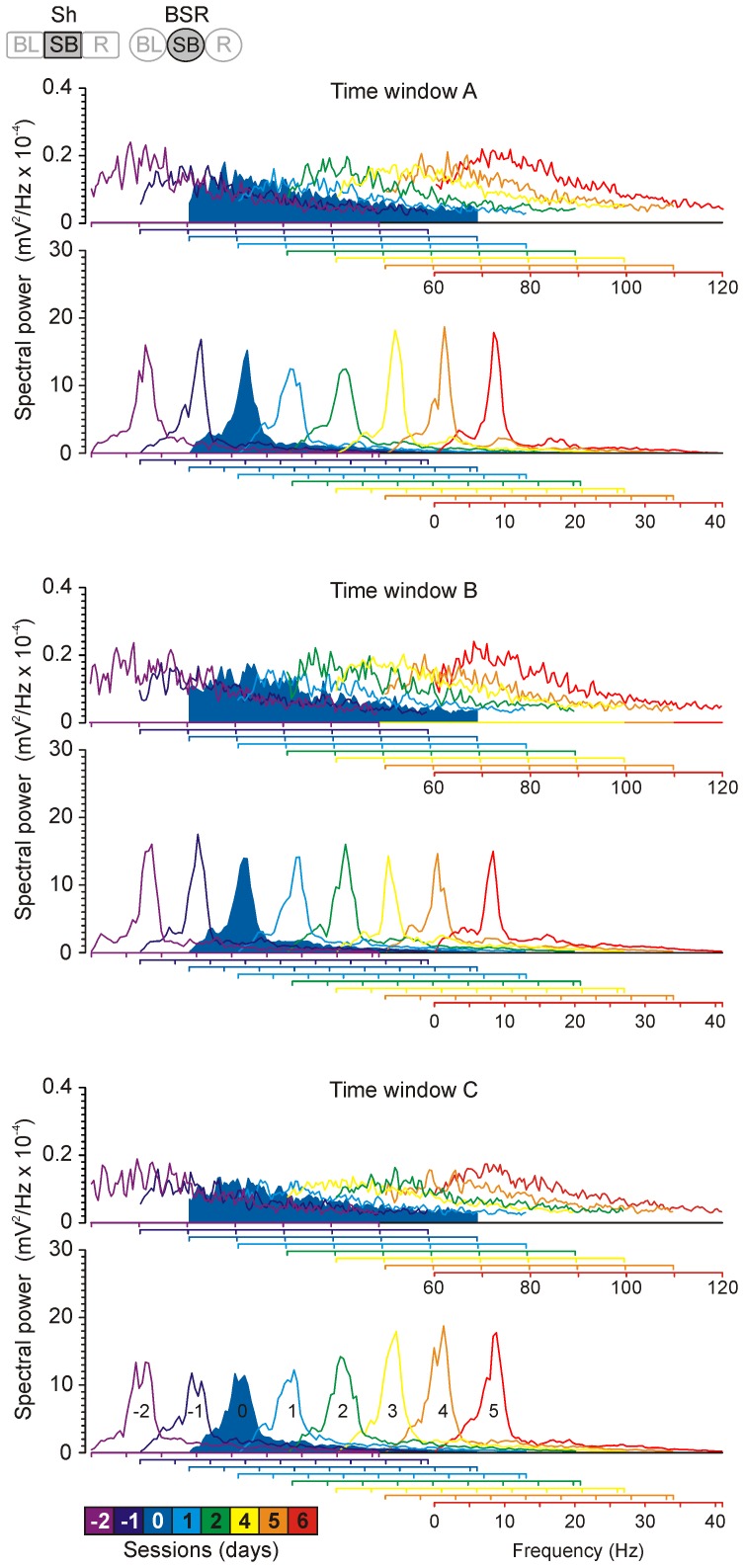
Spectral power analysis of EEGs recorded in the hippocampal CA1 area during shaping sessions and BSR performance. Data collected from a representative mouse during the previously defined time windows (A, B, and C) were analyzed each day along shaping and BSR protocols. From top to bottom, the three time windows are represented in three panels by semi-overlapped averaged spectral power profiles. The upper section of each panel represents high frequencies (60–120 Hz) and the bottom one the lower frequencies (1–40 Hz) during shaping (−2, −1 and 0) and brain stimulation (1–6) sessions. The session in which criterion was reached (day 0) is represented in solid blue. The color code corresponding to each illustrated session (from −2 to 6) is illustrated at the bottom. Code bars at the top are defined in Figure 1.

According to the present results, there is a negative trend in fEPSP amplitudes across BSR sessions, an occurrence not observed for fIPSP values (Figure 2C, D). These results could be a reflection of a similar mechanism previously described with regard to hippocampal rhythmic activities. This suggestion is in agreement with previous studies describing the relationship between fPSPs and theta rhythm—considering a complete band of 3–12 Hz. In 1990, Núñez and colleagues [71] reported that the participation of fEPSPs is important in the generation of the theta rhythm, whereas that of fIPSPs is not so necessary. Furthermore, in 2000, Wyble and colleagues, recording in CA1, reported a significant suppression of evoked fEPSP (single pulse in CA3 or dentate gyrus) when theta rhythm in the dentate gyrus was the dominant frequency (>75%). For this, they evaluated the power spectra density in epochs lasting three seconds prior to the stimulation [72]. Finally, in 2002 Seager and colleagues reported a facilitatory effect of theta rhythm during classical conditioning [68]. Although there are some differences in the experimental procedures, we can speculate that a similar relationship is reproduced in our results. We noticed a progressive decrease in fEPSP amplitudes across BSR sessions, a fact that could facilitate the observed increase in the power of the theta band. In addition, the decrease of fEPSP amplitudes was accompanied by an increasing trend of the main peak in the spectral power within the theta band (around 8–9 Hz) in time window C (Figure 7).
